# S100A14 Stimulates Cell Proliferation and Induces Cell Apoptosis at Different Concentrations via Receptor for Advanced Glycation End Products (RAGE)

**DOI:** 10.1371/journal.pone.0019375

**Published:** 2011-04-29

**Authors:** Qing'e Jin, Hongyan Chen, Aiping Luo, Fang Ding, Zhihua Liu

**Affiliations:** State Key Laboratory of Molecular Oncology, Cancer Institute, Chinese Academy of Medical Sciences and Peking Union Medical College, Beijing, China; Roswell Park Cancer Institute, United States of America

## Abstract

S100A14 is an EF-hand containing calcium-binding protein of the S100 protein family that exerts its biological effects on different types of cells. However, exact extracellular roles of S100A14 have not been clarified yet. Here we investigated the effects of S100A14 on esophageal squamous cell carcinoma (ESCC) cell lines. Results demonstrated that low doses of extracellular S100A14 stimulate cell proliferation and promote survival in KYSE180 cells through activating ERK1/2 MAPK and NF-κB signaling pathways. Immunoprecipitation assay showed that S100A14 binds to receptor for advanced glycation end products (RAGE) in KYSE180 cells. Inhibition of RAGE signaling by different approaches including siRNA for RAGE, overexpression of a dominant-negative RAGE construct or a RAGE antagonist peptide (AmphP) significantly blocked S100A14-induced effects, suggesting that S100A14 acts via RAGE ligation. Furthermore, mutation of the N-EF hand of S100A14 (E39A, E45A) virtually reduced 10 µg/ml S100A14-induced cell proliferation and ERK1/2 activation. However, high dose (80 µg/ml) of S100A14 causes apoptosis via the mitochondrial pathway with activation of caspase-3, caspase-9, and poly(ADP-ribose) polymerase. High dose S100A14 induces cell apoptosis is partially in a RAGE-dependent manner. This is the first study to demonstrate that S100A14 binds to RAGE and stimulates RAGE-dependent signaling cascades, promoting cell proliferation or triggering cell apoptosis at different doses.

## Introduction

S100 proteins are small calcium-binding proteins of the EF hand motif which can function as both intra- and extracellular signaling molecules. They exert a broad range of intracellular functions through the modulation of their subcellular localization and interacting with specific target proteins, responsible for cell growth, differentiation, motility, and cell cycle regulation [1], [2]. Some members are also secreted from cells exerting cytokine-like paracrine or autocrine functions although the precise mechanisms of secretion are still being elucidated [3]–[5]. Some S100 proteins added to the extracellular medium result in the translocation of the corresponding endogenous proteins [6]. Recently, S100 proteins became of major interest owing to their close association with several diseases including inflammation, neurodegenerative disorders and cancer [7], [8].

RAGE is a multiligand receptor of the immunoglobulin superfamily and is constitutively expressed during embryonic development, but its expression is down-regulated in adult life in physiological states [9]. RAGE binds to multiple families of ligands, such as advanced glycation end products (AGEs), S100s, and amphoterin, and plays a key role in diabetes, inflammation, and cancer [5], [10]. The cytoplasmic region of RAGE appears to be essential for RAGE signaling. RAGE ligation is known to activate multiple signaling pathways such as MAPK, JNKs, Cdc42/Rac, together with activation of transcription factors AP-1, NF-κB, etc. that regulate important cellular functions [2], [11], [12]. Several S100 family members are found in the extracellular medium and appear to have extracellular roles [7], [13]. For instance, S100B secreted by astrocytes activates the PI3K/AKT and NF-κB pathways via the engagement of RAGE, modulating cell survival [14]. S100A8/A9 has been shown to stimulate cell proliferation via p38MAPK and ERK1/2 activation in a RAGE dependent manner [15], [16]. Of course, there also are other receptors in addition to RAGE mediating biologic effects of S100 proteins. S100B causes myoblast apoptosis or inhibits myogenic differentiation in a RAGE-independent manner [17], [18]. S100A8/9 induces cell death that involves selective release of Smac/DIABLO and Omi/HtrA2 via a RAGE-independent pathway [19]. But to our knowledge, no data are available on the extracellular effect of S100A14.

S100A14 is a member of the S100 family of calcium-binding protein, whose biological function is largely unknown by now. It is differentially expressed in a wide variety of cell types and is up-regulated in certain types of tumors, such as lung, breast, and uterus, but down-regulated in some other tumors, such as colon, kidney, and rectal tumors [20]. S100A14 low-expression together with S100A4 high-expression was correlated with high metastatic potential in colorectal cancer [21]. S100A14 has been reported to be able to interact with nucleobindin (Calnuc) in a yeast two-hybrid system, a Golgi calcium binding protein which plays a key role in the constitution of calcium storage [22]. The significance of the translocation of S100A14 from cytosol to plasma membrane in breast cancer remains to be established [23]. S100A14 may play vital roles in bladder tumorigenesis and progression [24]. *S100A14* gene was found useful for detection of circulating tumor cells (CTCs) in peripheral blood of advanced cancer patients [25]. However, the functional role of S100A14 protein has not been identified yet. Our previous studies showed that *S100A14* gene was regulated by p53 and was associated with esophageal squamous cell carcinoma *in vivo* [26]. These evidences indicate the potential importance of this protein in tumors. We also found that S100A14 could be secreted from stably overexpressing S100A14 of EC9706 cells which did not show endogenous expression and KYSE180 cells which showed endogenous expression (See Figure S1). So we hypothesized that S100A14 might also exert extracellular roles.

In the present study, we investigated the function of exogenous S100A14 on ESCC cell lines. We found that low doses of exogenous S100A14 activate ERK1/2 and NF-κB signaling, stimulating cell proliferation or promoting cell survival via RAGE ligation; while high dose of S100A14 triggers apoptosis and increases the production of ROS also in a RAGE-dependent manner. Blocking the interaction of S100A14 and RAGE could inhibit the effects of S100A14 *in vitro*; therefore, exogenous S100A14 stimulates cell proliferation or induces apoptosis at different concentrations via RAGE ligation.

## Results

### Low Doses of Extracellular S100A14 Stimulate Cell Proliferation and Survival

**Figure 1:**
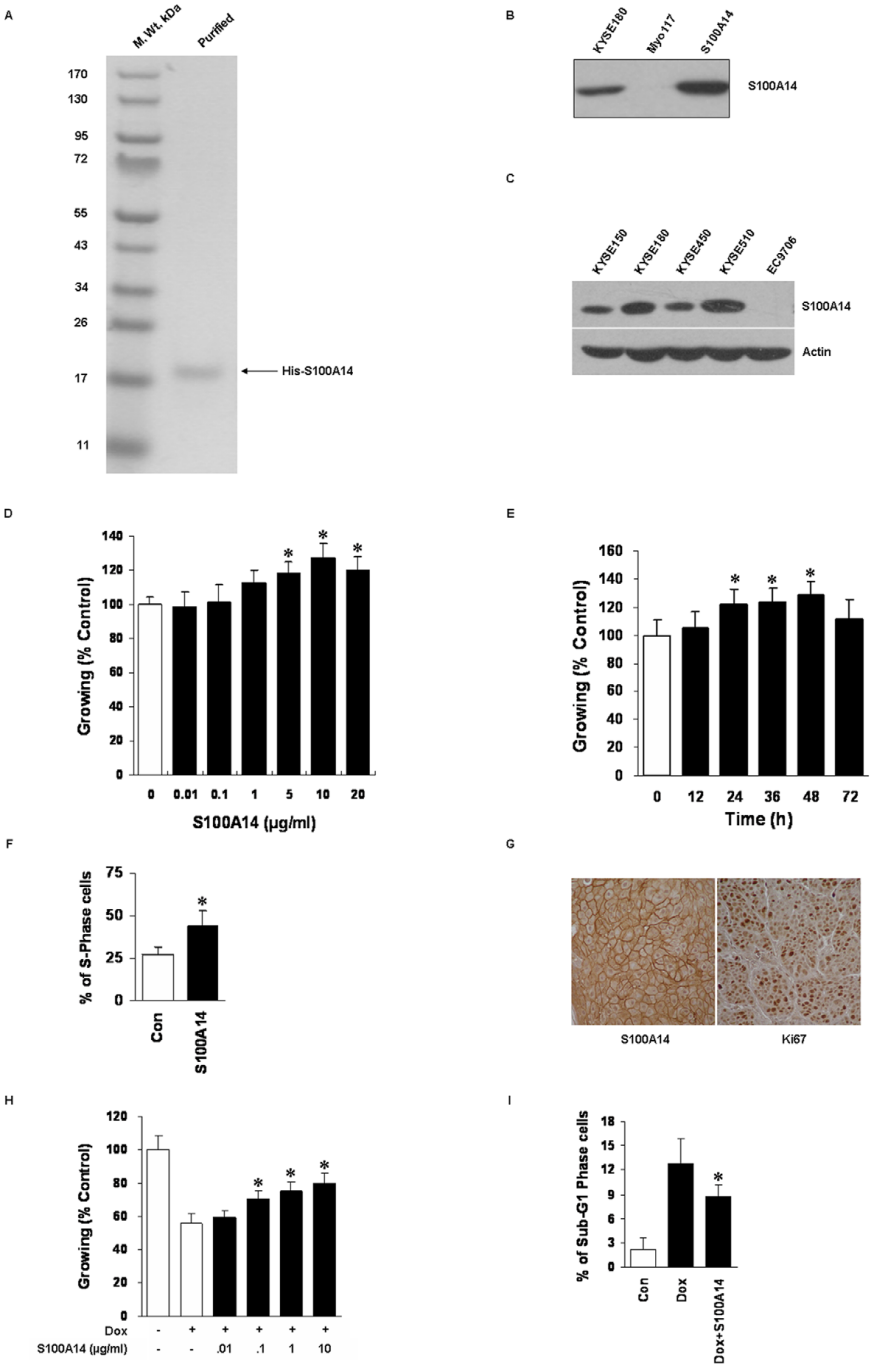
Low doses of S100A14 stimulate cell proliferation and promote cell survival. (**A**) Purity of S100A14 recombinant protein was analyzed by SDS-PAGE. (**B**) Specificity of S100A14 was tested by Western blot using S100A14 antibody. KYSE180 cell lysates were used as a positive control, and Myo117 protein was used as a negative control. (**C**) Endogenous S100A14 expression in ESCC cell lines was tested by Western blot. (**D**) S100A14-stimulated cell proliferation was dose-dependent. KYSE180 cells were incubated with indicated doses of S100A14 for 48 h. (**E**) S100A14-stimulated cell proliferation was time-dependent. At the dose of 10 µg/ml, evident effects were achieved at 48 h. (**F**) 10 µg/ml S100A14 increased S-phase percentage of KYSE180 cells at 48 h. (**G**) Immunohistochemical staining of S100A14 and Ki67 in ESCC specimens (×40). (**H**) Exogenous S100A14 increased cell survival exposed to Dox (0.5 µM, 48 h) in a dose-dependent manner. (**I**) 10 µg/ml S100A14 decreased the percentage of sub-G1 phase of KYSE180 cells with the treatment of Dox (0.5 µM, 48 h). Myo117 was used as a control. Cell viability was estimated using MTT assay and S-phase and sub-G1 phase were analyzed by flow cytometry assay. Results are expressed as difference to corresponding controls and represent the mean ± SD of three independent experiments (*, P<0.05).

To investigate the putative effect of extracellular S100A14 on cell function, we produced purified S100A14 as a histidine-tagged fusion protein in *Escherichia coli*. To ensure that the observed effects were caused by S100A14, we used Myo117 as a negative control, which is a protein of similar size, bearing the same His-tag and produced in an identical manner with S100A14 [27]. Purity and specificity of S100A14 protein were tested by SDS-PAGE and Western blot (Figures 1A and B). We screened five ESCC cell lines for the expression of S100A14 and finally chose KYSE180 cells which had relatively high level of S100A14 and EC9706 which had negligible endogenous S100A14 for further investigation (Figure 1C). Low doses of S100A14 (0.01–20 µg/ml) added to KYSE180 cells stimulated cell proliferation in a concentration- and time-dependent manner, with a remarkable increase at 5–20 µg/ml S100A14, as shown by MTT assay. The maximal effects were observed with 10 µg/ml S100A14, and evident increase in cell proliferation was noted within 48 h following 10 µg/ml S100A14 treatment (Figures 1D and E). The promotion in cell growth was further confirmed by FACS analysis, with an increase of S phase proportion of the cells treated with 10 µg/ml S100A14 for 48 h (Figure 1F). Similar effects were obtained from EC9706 cell line (data not shown), indicating that the effects were not confined to a single cell model. Additionally, immunohistochemical analysis of the expression of S100A14 and proliferation marker Ki67 was performed on forty-one paraffin-embedded ESCC specimens. S100A14 positivity significantly correlated with the expression of Ki67 (Figure 1G). The Spearman correlation coefficient was 0.440 (P = 0.004). The immunoreactivity and pathological characteristics of the specimens were shown in Table S1 and Table S2.

Extracellular S100A14 also increased the survival of KYSE180 cells exposed to the cytotoxic agent Doxorubicin (Dox). The addition of S100A14 protected cells from injuries induced by Dox (0.5 µM, 48 h) in a concentration-dependent manner with significant protection obtained at 10 µg/ml (Figure 1H). Furthermore, the survival benefits of S100A14 were confirmed by FACS analysis that 10 µg/ml S100A14 decreased the percentage of sub-G1 phase in KYSE180 cells with the treatment of Dox (Figure 1I).

### Impact of Low Doses of S100A14 on MAP Kinase and NF-κB

**Figure 2:**
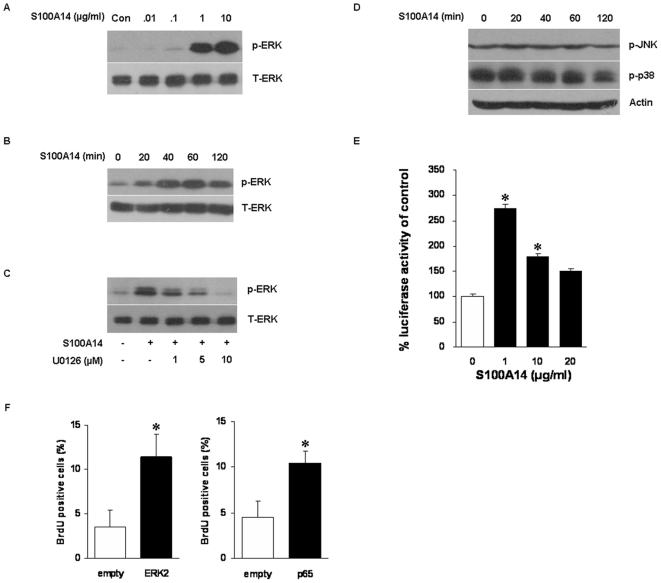
Low doses of S100A14 treatment activate ERK1/2 and NF-κB but not JNK or p38. (**A**) KYSE180 cells were incubated with different doses of S100A14 protein for 30 min, Myo117 was used as a control. (**B**) 10 µg/ml S100A14 added to KYSE180 cells for the indicated time periods. (**C**) Cells were pretreated with the MEK/ERK inhibitor U0126 for 30 min, followed by 10 µg/ml S100A14 exposure for another 30 min. T-ERK was used as a loading control. (**D**) No increased activity of the MAP kinase JNK or p38 was observed. Actin was used as a loading control. (**E**) Influence of S100A14 on NF-κB activity in KYSE180 cells was examined by luciferase reporter assay. Cells were treated with the indicated doses of S100A14 for 8 h and subjected to analysis of NF-κB activity (*, P<0.05). (**F**) KYSE180 cells transfected with ERK2 or NF-κB p65 plasmid were cultured for 24 h in the presence of 10 µM BrdU. Empty vector was transfected as a negative control. The percentage of BrdU-positive cells over the total number of cells was determined (*, P<0.05). Bars represent mean ± SD of three independent experiments.

We next determined whether the effects of S100A14 on cell proliferation and survival were related with the common signal pathways. Consistent with cell growth, exogenous S100A14 induced ERK1/2 activation in a concentration- and time-dependent manner, with significant effect observed at 1 µg/ml and maximal effect achieved at 10 µg/ml with an incubation of 30 min (Figure 2A). With the concentration of 10 µg/ml, ERK1/2 activation was noted significantly within 40 min, and phosphorylation sustained up to 120 min (Figure 2B). To verify the involvement of MAPK pathway, KYSE180 cells were then treated with S100A14 (10 µg/ml, 30 min) in the presence of different doses of U0126, an inhibitor of MEK/ERK. As shown in Figure 2C, U0126 drastically reduced the activation of ERK1/2, in contrast, the phosphorylation of SAPK/JNK and p38 remained unchanged with the treatment of 10 µg/ml S100A14 (Figure 2D).

Moreover, NF-κB activation is an important event mediating cell growth and survival, thus we analyzed NF-κB activity in KYSE180 cells transfected with NF-κB reporter plasmid which then treated with low doses of S100A14 for 8 h. As shown in Figure 2E, NF-κB activity was significantly activated with 1 µg/ml S100A14 for up to 275.5%. Contrast to the above results, 1 µg/ml rather than 10 µg/ml S100A14 evidently stimulated NF-κB activation.

We also found that constitutive activation of ERK or NF-κB could regulate cell proliferation as low doses of S100A14 in KYSE180 cells by BrdU assays (Figure 2F). Taken together, the study indicated that low doses of S100A14 stimulated cell proliferation and survival through the activation of p44/42 MAPK and NF-κB signaling pathways.

### S100A14 Functions via RAGE Activation

**Figure 3:**
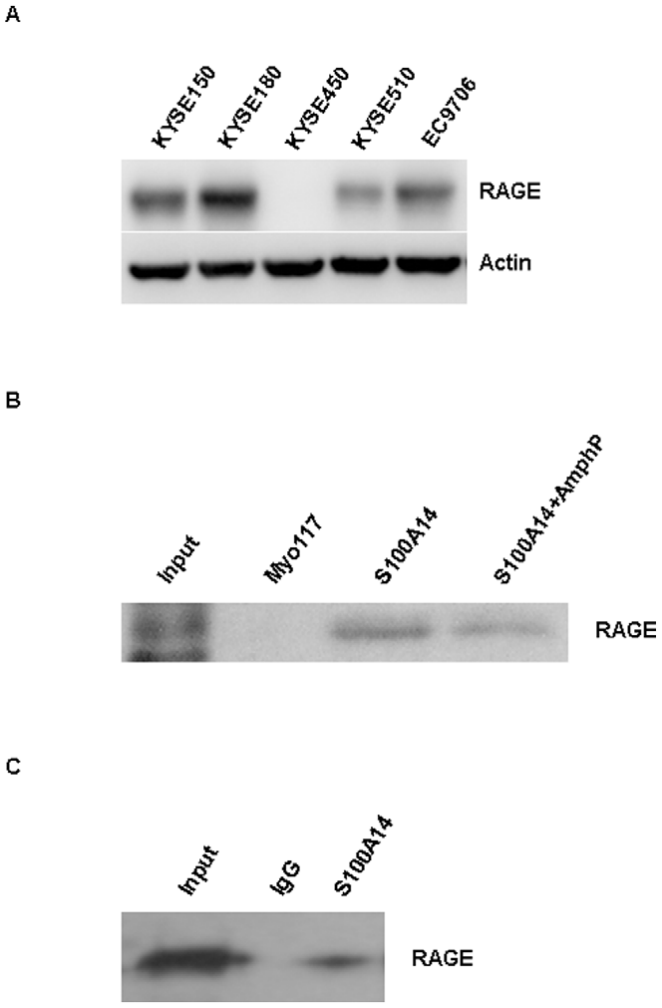
The interaction of S100A14 with RAGE. (**A**) Western blot showed the endogenous RAGE expression in ESCC cell lines. (**B**) KYSE180 cell lysates were incubated with exogenous S100A14 and His-resin. RAGE was identified in the immunoprecipitates by Western blot with an anti-RAGE antibody. KYSE180 cell lysates were used as a control. Myo117 protein was added as a negative control. Preincubation with AmphP (5 µM) for 1 h interfered with the interaction of S100A14 and RAGE. (**C**) KYSE180 cell lysates were immunoprecipitated with anti-S100A14 polyclonal antibodies and RAGE was identified in the immunoprecipitates by Western blotting.

RAGE ligation can activate multiple signaling pathways. Previous studies revealed that several S100 family members can function extracellularly through RAGE ligation [3]. So we presume that RAGE could be the receptor responsible for S100A14-induced cell effects. To test the hypothesis, we first examined the expression of RAGE in ESCC cell lines, and found that RAGE can be detected in four of five ESCC cell lines except for KYSE450 cells (Figure 3A). Immunofluorescence assay showed that RAGE was mainly localized on the cell surface of four ESCC cell lines (data not shown). Pull-down assay was then performed using KYSE180 lysates with purified S100A14 to investigate whether there is a direct interaction between S100A14 and RAGE. Result indicated that S100A14 could bind to RAGE from KYSE180 cell lysates. Furthermore, the interaction can be inhibited by preincubation with a RAGE antagonist peptide (AmphP) [28], which had been reported to block the interaction of RAGE and ligands (Figure 3B). To further confirm the interaction, we performed co-immunoprecipitation assays using lysates from KYSE180 cells. RAGE was identified in the precipitate, indicating an interaction between S100A14 and RAGE (Figure 3C).

**Figure 4:**
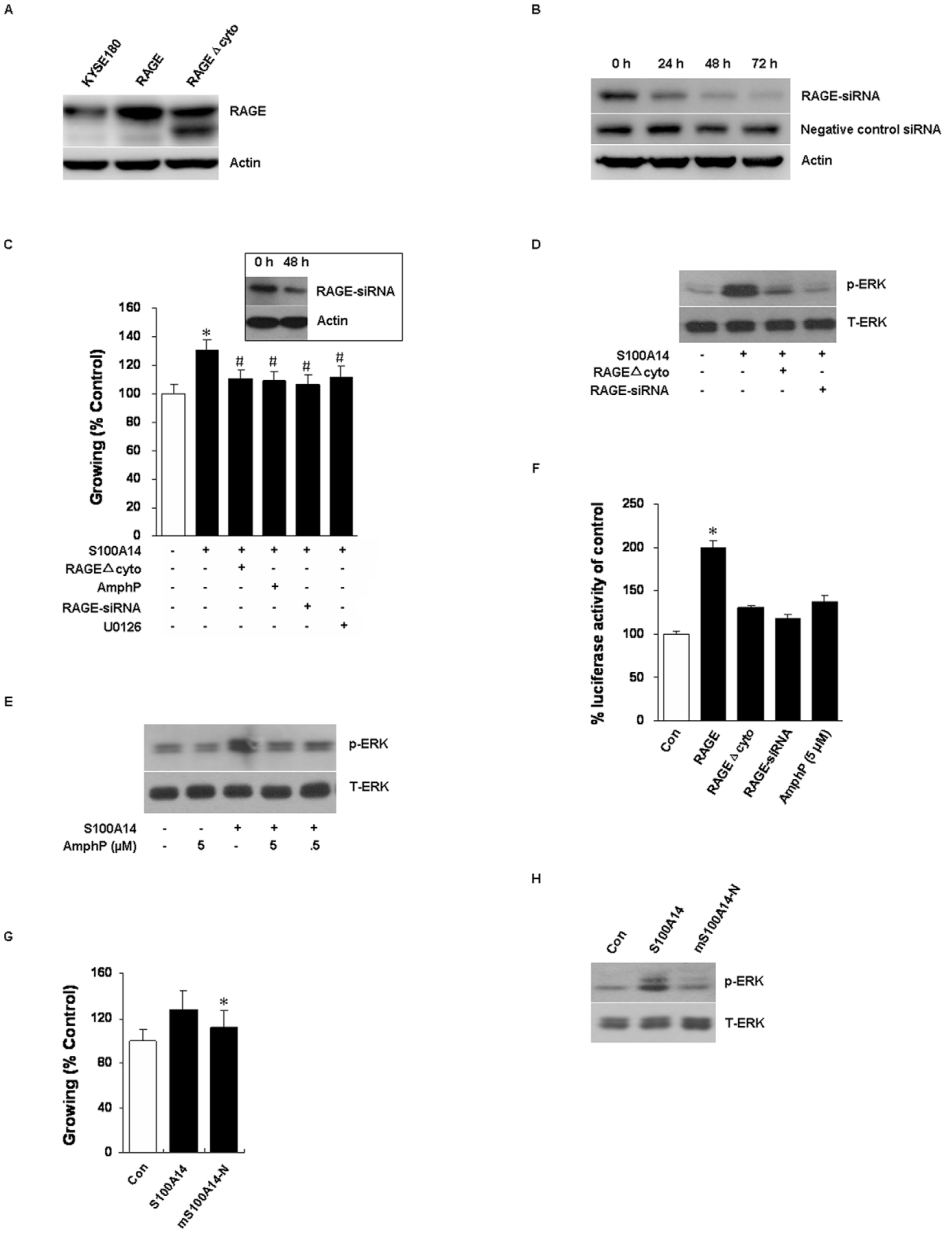
The effects of S100A14 were dependent on RAGE ligation. (**A**) Overexpression of RAGE and RAGEΔcyto in KYSE180 cells were confirmed by Western blot. (**B**) siRNA-mediated RAGE silencing in KYSE180 cells for indicated intervals was detected by Western blot. (**C**) KYSE180 or KYSE180-RAGEΔcyto cells were treated with 10 µg/ml S100A14 (+) or Myo117 (−) and with (+) or without (−) 5 µM AmphP or 50 nM RAGE-siRNA or 5 µM U0126 for 48 h, and the effects on cell proliferation were analyzed by MTT assays (*, P<0.05 *versus* control; #, P<0.05 *versus* S100A14). Western blot showed the effect of RAGE silence at 48 h. (**D**) KYSE180 or KYSE180-RAGEΔcyto or KYSE180-RAGE-siRNA cells were treated with 10 µg/ml S100A14 (+) or Myo117 (−) for 30 min, and the effects on ERK1/2 activation were estimated by Western blot. T-ERK was served as a loading control. (**E**) S100A14-induced ERK1/2 activation was inhibited by AmphP. KYSE180 cells were incubated with 10 µg/ml S100A14 (+) or different doses of AmphP for 30 min. (**F**) KYSE180-RAGE or KYSE180-RAGEΔcyto cells were treated with 10 µg/ml S100A14 (+) or Myo117 (−) or RAGE-siRNA or AmphP for 8 h, then NF-κB activity was examined by luciferase reporter assay (*, P<0.05). (**G**) mS100A14-N protein (mutation of N-EF hand amino acid, E39A, E45A) can not stimulate cell proliferation (*, P<0.05 *versus* S100A14). (**H**) mS100A14-N can not activate ERK1/2 in KYSE180 cells. Bars represent mean ± SD of three independent experiments.

We subsequently constructed KYSE180 cell clones stably expressing either full-length RAGE or dominant-negative mutant of RAGE which were designated as KYSE180-RAGE or KYSE180-RAGEΔcyto, respectively (Figure 4A). Meanwhile, RAGE expression was suppressed in KYSE180 cells by RAGE-specific siRNA, as shown in Figure 4B.

To explore whether RAGE was involved in cell proliferation and the signaling pathways driven by S100A14, we utilized several methods to inhibit RAGE activation. Overexpression of RAGEΔcyto or addition of AmphP or pretreatment with RAGE-siRNA or incubation with U0126, all these treatments significantly inhibited 10 µg/ml S100A14-induced KYSE180 cell proliferation (Figure 4C). Meanwhile, inhibition of RAGE function by overexpression of RAGEΔcyto or pretreatment with RAGE-siRNA evidently blocked ERK1/2 activation induced by 10 µg/ml S100A14 (Figure 4D). Likewise, preincubation with AmphP also inhibited S100A14-induced ERK1/2 activation (Figure 4E). KYSE180-RAGE or KYSE180-RAGEΔcyto transiently transfected with NF-κB reporter plasmid was subsequently stimulated with 10 µg/ml S100A14 for 8 h. KYSE180-RAGE cells showed a strong induction of NF-κB dependent transcriptional activity, whereas KYSE180-RAGEΔcyto cells did not show significant changes to extracellular S100A14. The same results were found in KYSE180 cells pretreatment with RAGE-siRNA or addition of AmphP before stimulation with S100A14 (Figure 4F). The above experiments were also performed in KYSE450 cells stably transfected RAGE or RAGEΔcyto plasmid which did not express RAGE endogenously, and similar results were obtained (data not shown).

Further studies indicated that key amino acid mutations of N-EF hand (E39A, E45A) significantly attenuated the effects of S100A14. Cell viability assays indicated that incubation with mS100A14-N protein (10 µg/ml, 48 h) did not stimulate KYSE180 cell proliferation compared to those with S100A14 (Figure 4G). As expected, 30 min incubation of mS100A14-N protein also abrogated ERK1/2 activation (Figure 4H), indicating that key amino acid of N-EF hand might play an important role on S100A14 function. These results suggest that the interaction between RAGE and S100A14 may play an important role in cell proliferation and survival by activating ERK1/2 and NF-κB signaling.

### Impact of High Dose of S100A14 on Cell Viability

**Figure 5:**
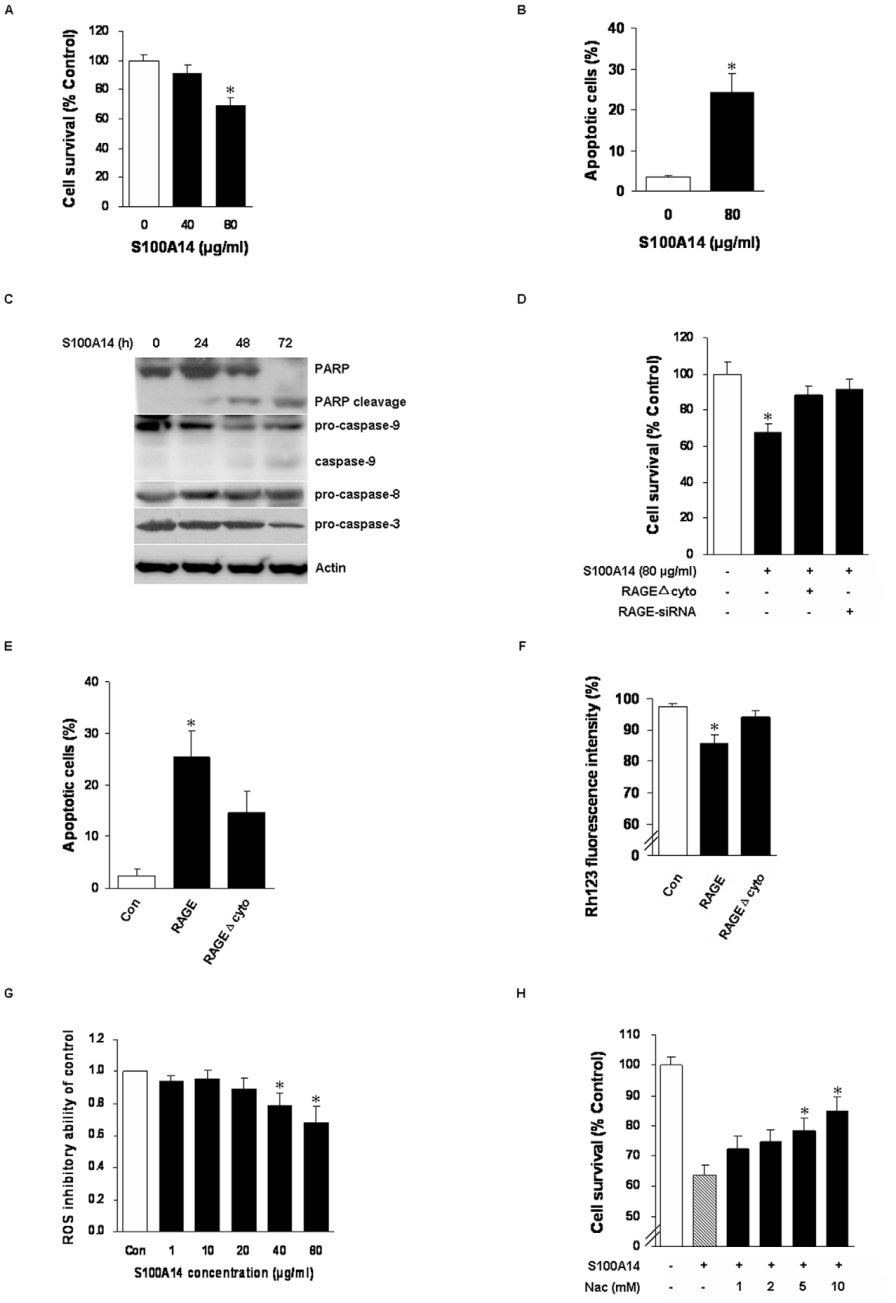
High dose of S100A14-induced apoptosis in KYSE180 cells is partially RAGE dependent. 80 µg/ml S100A14 treated KYSE180 cells for 48 h significantly triggered cell injuries as detected by MTT (**A**) and flow cytometry assay (**B**). (**C**) KYSE180 cells were treated with 80 µg/ml S100A14 for indicated periods, then caspase-3, caspase-8, caspase-9, PARP and Actin were detected by Western Blot. (**D**) Inhibition of RAGE function by RAGEΔcyto or RAGE-siRNA evidently blocked 80 µg/ml S100A14-induced cell death by MTT assay. (**E**) Compared to KYSE180-RAGE, KYSE180-RAGEΔcyto cells had more slightly injuries with 80 µg/ml S100A14 for 48 h as detected by Annexin V-PI staining assay. (**F**) Cells were stained with Rh123 before flow cytometry. Data revealed the decrease of mitochondrial membrane potential in KYSE180-RAGE in contrast to KYSE180-RAGEΔcyto treated with 80 µg/ml S100A14 for 48 h (*, P<0.05). (**G**) High doses of S100A14 induce the generation of ROS in KYSE180 cells. Cells were treated with different doses of S100A14 for 5 h and the supernatant were used to evaluate the production of ROS. Data are expressed as means ± SD of three independent experiments (*, P<0.05). (**H**) Inhibition of ROS production by Nac prevented cell injuries induced by 80 µg/ml S100A14. KYSE180 cells were preincubated with the antioxidant for 24 h then exposed to S100A14 for another 24 h, MTT assay was then performed. All values represent the means ± SD of three independent experiments (*, P<0.05).

In addition to cell proliferation, S100A14 was able to trigger cell injuries at relatively high dose. We found that 80 µg/ml S100A14 could remarkably induce cytotoxicity by evaluating the cell viability using MTT assay (Figure 5A). To investigate the underlying mechanisms, we detected the effect of S100A14-induced apoptosis and the activation of apoptotic mediators. Flow cytometry analysis showed that 80 µg/ml S100A14 significantly triggered cell apoptosis (Figure 5B). Western blot displayed the increased activity of apoptotic caspase-3, caspase-9, poly(ADP-ribose) polymerase, but pro-apoptotic caspase-8 was unchanged (Figure 5C), indicating that cell death was probably mediated by the mitochondrial apoptotic pathway. These data were in agreement with a report of Ghavami et al. demonstrating that S100A8/9 within the range of 80–100 µg/ml induced apoptosis in colon cancer cell lines [29]. We further investigated whether the effect was dependent on RAGE activation. As shown in Figure 5D, indeed 80 µg/ml S100A14 treatment of KYSE180-RAGEΔcyto or KYSE180-RAGE-siRNA cells significantly attenuated cell injuries in contrast to KYSE180 cells by MTT assay. Moreover, the apoptotic cells from KYSE180-RAGE were increased up to 25.4%, whereas those from KYSE180-RAGEΔcyto cells were increased to 14.7% as detected by flow cytometry assay (Figure 5E). These data suggested that apoptosis induced by S100A14 could be partially RAGE-dependent and RAGE inhibition afforded a protective effect from the toxicity of S100A14.

To further substantiate the data, mitochondrial membrane potential (MMP) was measured. As shown in Figure 5F, KYSE180-RAGE showed a significant reduction of the MMP compared with KYSE180-RAGEΔcyto cells and the mock control by the addition of 80 µg/ml S100A14 for 48 h, which points to mitochondrial dysfunction, an event commonly associated with ROS production and apoptosis [30]. Vincent et al. found that RAGE-induced PI3K activity was associated with the formation of ROS, caspase-3 activation and nuclear DNA degradation [31]. Therefore, we further investigated whether 80 µg/ml S100A14-induced apoptosis was related with the production of ROS. As shown in Figure 5G, high dose of S100A14 could induce the formation of ROS in KYSE180 cells and the antioxidant *N*-acetyl-L-cysteine (Nac) was capable of conferring protection against 80 µg/ml S100A14-induced cell injuries in a dose-dependent manner (Figure 5H), which confirmed that ROS was involved in the injury process induced by S100A14. While 80 µg/ml S100A14 could not result in increased production of ROS in KYSE180-RAGEΔcyto cells indicating that S100A14-induced ROS formation was also RAGE dependent (data not shown).

## Discussion

More attention has been paid to the extracellular functions of S100 proteins recent years. So far, the S100 family members S100A4, S100A6, S100A8/9, S100A12, S100A13, S100B and S100P have been shown to interact with RAGE, resulting in activation of signal pathways and involving in numerous pathologic situations [3], [13]. S100B and S100A12 were the first members of the family found to interact with RAGE and induce cellular effects [32]. Extracellular S100A4 stimulates matrix metalloproteinase 13 release from chondrocytes in a RAGE-mediated manner [33]. S100A11 has previously been shown to activate RAGE signaling via a p38 MAPK pathway, promoting chondrocytes hypertrophy [34]. Exogenous S100P stimulates cell proliferation, migration, invasion, and activates MAP kinase and NF-κB pathways via RAGE ligation in pancreatic and colon cancer cell lines [35]–[37]. Inhibition of the interaction and signaling of RAGE and ligands could suppress tumor growth, motility and metastasis in nude mice [11].

We have found that S100A14 was secreted into the extracellular medium from *S100A14* transfected EC9706 stable clones and KYSE180 cells, and extracellular S100A14 might regulate its endogenous expression, creating a positive feedback loop. However, it is unclear whether S100A14 exerts extracellular effects. In the present study, using human ESCC cell lines, we found for the first time that low doses of S100A14 promote cell proliferation in a concentration- and time-dependent manner. S100A14 also protected KYSE180 cells from injuries induced by chemotherapeutic agent Dox. At the intracellular level, at least two signal pathways ERK and NF-κB were involved. Low doses of S100A14 stimulate ERK1/2 phosphorylation in a concentration- and time-dependent manner. In contrast, the phosphorylation of JNK or p38 was unchanged which has been classically linked to cell stress and induction of apoptosis [38]. NF-κB is an important transcription factor linked to many signal pathways, activating the expression of various molecules that mediate many biological events including cell survival. Luciferase assay indicated that NF-κB signaling was also involved in the process.

More importantly, we have confirmed the direct interaction of S100A14 and RAGE by pull-down and co-immunoprecipitation assays, and further shown that S100A14 stimulated KYSE180 cell proliferation via interaction with RAGE, since the addition of siRNA for RAGE, AmphP, or RAGEΔcyto expression were all able to clearly block the proliferation effect in a concerted manner. The effects were similar with that of S100P on NIH3T3 cells, which may due to considerable sequence homology within S100 protein family members [35]. Previously it has been shown that key amino acid mutation of EF hand of S100A4 is responsible for metastasis-inducing properties and self-association [39], [40]. To explore the role of amino acid of EF hand of S100A14, we produced mutant protein. As shown in Fig. 4G and H, mutations of the N-EF hand of S100A14 (E39A, E45A) virtually abolished its effects on cell proliferation and ERK1/2 activation, suggesting that N-EF hand motif of S100A14 was presumably responsible for RAGE binding and function.

Another aspect of the cellular function of S100 proteins is related with cell death. For example, S100B was found to exert a cytotoxic effect via stimulation of ROS production in myoblast [17]. In human SH-SY5Y neuroblastoma cells, S100A6 bound to RAGE triggers the JNK and caspase pathways, resulting in apoptosis [14]. Sustained activation of ERK1/2 resulting in excessive RAGE-dependent production of ROS has been proposed as the mechanism of high concentration of S100B-induced neuronal death [41]. Interestingly, in the current study, we have demonstrated that treatment of KYSE180 cells with high dose of S100A14 also leads to cell apoptosis, displaying a PARP and caspase-9 cleavage, as well as a reduction of the mitochondrial membrane potential. Nac, an inhibitor of ROS, inhibited the injuries induced by high dose of S100A14, indicating that S100A14-induced apoptosis likely depended on intracellular accumulation of ROS.

Furthermore, the cytotoxic activity of high dose S100A14 is also RAGE-dependent since KYSE180 cells transfected with RAGE siRNA or expressing RAGEΔcyto both remarkably inhibited its toxic effects, and KYSE180-RAGEΔcyto obviously protected cells from the decrease of mitochondrial membrane potential. RAGE knockdown, at least in part, reversed the injury of high dose of S100A14, which was different from S100A8/9 whose bimodal function is mediated by two distinct receptors and signaling pathways [16], [19]. Moreover, we also observed that S100A14 can not promote RAGE-negative KYSE450 cell proliferation; while it can again stimulate cell proliferation and activate ERK1/2 on KYSE450 cells by overexpressing full-length RAGE (data not shown).

In summary, the work presented in this paper revealed important dual effects of S100A14 on KYSE180 cells. We have for the first time demonstrated that S100A14 can interact with RAGE, activating ERK1/2 and NF-κB signaling, promoting cell proliferation and survival at relatively low doses; whereas high dose of S100A14 induced apoptosis in a RAGE-mediated and oxidant-dependent manner. Our data are in line with the function of S100B with trophic or toxic effects on neurons or neuroblastoma cells depending on its concentration via RAGE ligation [41], [42]. An increasing body of evidence suggests that RAGE can function in other types of tumor cells. However, little is known concerning the function of RAGE in ESCC cell lines. The current data suggest that RAGE activation may be crucial for the effects of exogenous S100A14. However, it remains possible that there are other mechanisms or receptors participating in the process since inhibition of RAGE function can only block the effects partially and further study will be necessary to fully understand the complex biological behavior of S100A14 on ESCC cells *in vitro* and *in vivo*. Moreover, investigation of S100A14 signaling, regulation of S100A14 expression and secretion as well as interacting proteins may help unveil the complicated biological process. Given the prominent role of S100A14 on cell proliferation and apoptosis, therapeutic intervention targeting the S100A14 and RAGE signaling pathway may provide a novel approach for cancer therapy.

## Materials and Methods

### Expression and Purification of S100A14 Protein

Expression and purification of the human recombinant S100A14 protein in *E. coli* was performed as described [35]. After dialysis against PBS (pH 7.4) containing 0.1% Triton X-100 overnight at 4°C using dialysis membrane (Spectrum), the protein was concentrated using PEG 20000. Control plasmid of Myo117 with the similar size of S100A14 was a gift from Dr. Mariam Grigorian (Institute of Cancer Biology, Copenhagen, Denmark) to monitor for nonspecific effects which was expressed and purified in an identical manner [27].

### Site-directed Mutagenesis of S100A14

Point mutations were introduced into the *S100A14* cDNA to convert calcium-coordinating amino acids in the loop of the N-EF hand E39, E45 to A using site-directed mutagenesis by PCR (Beijing SBS), which was designated as mS100A14-N. The plasmid pET-28a-S100A14 was used as template. The nucleotide sequences of the mutant cDNA were checked by DNA sequencing (Applied Biosystems).

### Cell Culture, Treatment and RNA Interference

Human ESCC cell lines KYSE180 and KYSE450 were gifts from Dr. Y. Shimada of Kyoto University (Kyoto, Japan) [43], and EC9706 was established in our own laboratory [44]. Cells were cultured in RPMI 1640 medium supplemented with penicillin/streptomycin (100 units/ml), and 10% fetal bovine serum at 37°C in 5% CO_2_ humidified atmosphere. After plating, cells were cultivated for 24 h and then serum-starved for an additional 24 h before treatment with indicated doses of S100A14 in serum-free medium. The target siRNA for RAGE (sc-36374) and a negative-control siRNA (sc-37007) with an irrelevant sequence were purchased from Santa Cruz Biotechnology (Santa Cruz). Cells were grown to 60% confluence and then transfected with the siRNA duplex (50 nM) using HiperFect (Qiagene) according to the manufacturer's instructions. The transfected cells (72 h post-transfection) were then treated with S100A14 for indicated periods.

### Establishment of Stable Cell Lines

Cells were seeded at 35-mm-diameter plate and transfected using Lipofectamine 2000 (Invitrogen) with pcDNA3.0/RAGE and pcDNA3.0/RAGEΔcyto plasmids [12]. Stable cell lines were established following selection with 400 µg/ml G418 (Life Technologies).

### Cell Viability Assays

Different concentrations of S100A14 were added to the cells for indicated time intervals, and cell proliferation was determined by MTT assay as instructed by the manufacturer (Roche Applied Science). Percentage of viability was determined by comparing the number of viable cells in treated culture to that in control culture treated in parallel with Myo117 protein. For survival test, cells were treated with 0.5 µM Dox as well as S100A14 protein for 48 h, cell viability was then estimated by MTT assay.

Incorporation of 5-bromo-2′-deoxyuridine (BrdU) was performed as another cell proliferation assay according to the manufacturer's protocol (Roche Applied Science).

### Immunoblotting

Protein extraction and Western blot were performed as described [45]. Antibodies for MAPKs, PARP, caspase-3, caspase-8 and caspase-9 were from Santa Cruz. Antibody for RAGE was from R&D Systems, Inc. β-actin antibody (Sigma, A5316) was used to test for equal loading. Antibody against S100A14 was a generous gift from Dr. Iver Petersen (University Hospital Charite', Berlin, Germany). ESCC cells were treated with different doses of S100A14 for different intervals.

For S100A14-RAGE pull-down experiments, purified S100A14 protein (10 µg) was incubated with His-resin beads at 4°C for 1 h and then incubated with KYSE180 cell lysates overnight at 4°C. In some experiments, cells were pretreated with AmphP for 1 h before incubated with KYSE180 lysates. Bound proteins were eluted with 2×SDS sample buffer and subjected to SDS-PAGE, followed by immunoblotting using a RAGE polyclonal antibody.

For coimmunoprecipitation experiments, KYSE180 cell lysates were immunoprecipitated with anti-S100A14 antibody at 4°C overnight and RAGE was identified in the immunoprecipitates by Western blotting.

### Immunohistochemical Staining

Paraffin-embedded forty-one ESCC samples were preserved in our lab [45]. As for antigen retrieval, we used citrate buffer (pH 6.0) boiling for 15 minutes at microwave. Sections were incubated with primary antibody (mouse anti human Ki67 from Santa Cruz and S100A14 from Dr. Iver Petersen) at a 1∶100 dilution overnight at 4°C. DAB was used to visualize the reaction, followed by counterstaining with Hematoxylin.

### Flow Cytometry Assays

Cells were seeded onto 35-mm plastic dishes for 24 h, and cultivated for 48 h in the presence of different doses of protein. Standard PI staining was used for cell cycle analysis and the content of sub-G1 was regarded as apoptotic cells. Annexin V-PI staining was used for the apoptosis assay [46]. Annexin V was incubated for 30 min and PI for 5 min according to the manufacturer's protocol (Beijing Biosea) for the analysis of apoptotic cells.

### Luciferase Reporter Assays

Cells (approx.1.0×10^4^) were seeded in a 96-well plate and transfected with the NF-κB reporter plasmid using Lipofectamine 2000 (Invitrogen). 24 h after transfection, indicated doses of S100A14 were added to the medium. Cells were harvested 8 h later and assayed for luciferase activity using the Dual Luciferase Reporter Assay System (Promega) according to the manufacturer's manual. The luciferase activities were normalized to the *Renilla* luciferase activity of the internal control. NF-κB activity is expressed as relative luciferase activity as compared to control cells.

### Determination of Mitochondrial Membrane Potential

To detect changes in the mitochondrial membrane potential, KYSE180 cells were incubated for 10 min at 37°C with 10 µM Rh123 RPMI 1640 working solution. Cells were washed three times in PBS and then resuspended in RPMI 1640. A total of 10,000 cells were measured per sample by FACS flow cytometer (Becton Dickinson).

### Intracellular ROS Formation Assays

Intracellular ROS production was evaluated by using indirect ROS inhibitory kit (Nanjing Jiancheng Bioengineering Institute, China) according to the manufacturer's protocol. The clear ability of superoxide is high in untreated control cells considering as 100%, while increased intracellular ROS caused decreased clear capacity.

### Statistical Analysis

Data were presented as mean ± SD and one-way analysis of variance (ANOVA) analysis was performed using SPSS 13.0 software. The correlation between S100A14 and Ki67 was assessed using Spearman correlation analysis. P<0.05 was considered as significant.

## Supporting Information

Figure S1 **Western blot analysis for secretion of S100A14 from EC9706 or KYSE180 cells.** (**A**) Western blot was performed to detect S100A14 in the culture media from EC9706 cells stably transfected with HA-tag S100A14 full-length plasmid. Stable EC9706 transfectants with empty vector were used as a negative control (Mock). (**B**) S100A14 was secreted extracellularly from KYSE180 cells whereas no S100A14 secretion could be detected in EC9706 cells.
(DOC)

Table S1 **Immunoreactivity for S100A14 and Ki67 in 41 ESCC specimens.** For each case, an immunostaining score was given based on the percentage of cells showing definitive staining regardless of the staining intensity: 0 =  no staining, 1 =  less than 10%, 2 = 10–25%, 3 = 26–50%, 4 = >50% of cells stained.
(DOC)

Table S2 **The pathological characteristics of the specimens.**
(DOC)
