# Supplementary material for: S100A14 Stimulates Cell Proliferation and Induces Cell Apoptosis at Different Concentrations via Receptor for Advanced Glycation End Products (RAGE)
Source: PLoS One. 2011 Apr 29;6(4):e19375. doi: 10.1371/journal.pone.0019375 (PMC3084824; doi:10.1371/journal.pone.0019375)
Supplement: Figure S1 — Western blot analysis for secretion of S100A14 from EC9706 or KYSE180 cells. (A) Western blot was performed to detect S100A14 in the culture media from EC9706 cells stably transfected with HA-tag S100A14 full-length plasmid. Stable EC9706 transfectants with empty vector were used as a negative control (Mock). (B) S100A14 was secreted extracellularly from KYSE180 cells whereas no S100A14 secretion could be detected in EC9706 cells. (DOC) [file pone.0019375.s001.doc]

**Figure S1.** **Western blot analysis for secretion of S100A14 from EC9706 or KYSE180 cells.**

**(A)** Western blot was performed to detect S100A14 in the culture media from EC9706 cells stably transfected with HA-tag S100A14 full-length plasmid. Stable EC9706 transfectants with empty vector were used as a negative control (Mock). **(B)** S100A14 was secreted extracellularly from KYSE180 cells whereas no S100A14 secretion could be detected in EC9706 cells.
