# Supplementary material for: S100A14 Stimulates Cell Proliferation and Induces Cell Apoptosis at Different Concentrations via Receptor for Advanced Glycation End Products (RAGE)
Source: PLoS One. 2011 Apr 29;6(4):e19375. doi: 10.1371/journal.pone.0019375 (PMC3084824; doi:10.1371/journal.pone.0019375)
Supplement: Table S1 — Immunoreactivity for S100A14 and Ki67 in 41 ESCC specimens. For each case, an immunostaining score was given based on the percentage of cells showing definitive staining regardless of the staining intensity: 0 = no staining, 1 = less than 10%, 2 = 10–25%, 3 = 26–50%, 4 = >50% of cells stained. (DOC) [file pone.0019375.s002.doc]

**Table S1. Immunoreactivity for S100A14 and Ki67 in 41 ESCC specimens.**

For each case, an immunostaining score was given based on the percentage of cells showing definitive staining regardless of the staining intensity: 0 = no staining, 1 = less than 10%, 2 = 10-25%, 3 = 26-50%, 4 = > 50% of cells stained.
