# Supplementary material for: S100A14 Stimulates Cell Proliferation and Induces Cell Apoptosis at Different Concentrations via Receptor for Advanced Glycation End Products (RAGE)
Source: PLoS One. 2011 Apr 29;6(4):e19375. doi: 10.1371/journal.pone.0019375 (PMC3084824; doi:10.1371/journal.pone.0019375)
Supplement: Table S2 — The pathological characteristics of the specimens. (DOC) [file pone.0019375.s003.doc]

**Table S2. The pathological characteristics of the specimens.**
