# Supplementary figures and images for: Correction: S100A14 Stimulates Cell Proliferation and Induces Cell Apoptosis at Different Concentrations via Receptor for Advanced Glycation End Products (RAGE)
Source: PLoS One. 2016 Jan 22;11(1):e0147881. doi: 10.1371/journal.pone.0147881 (PMC4723040; doi:10.1371/journal.pone.0147881)

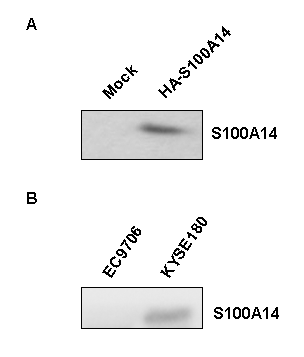

Supplement: S1 Fig — (A) Western blot was performed to detect S100A14 in the culture media from EC9706 cells stably transfected with HA-tag S100A14 full-length plasmid. Stable EC9706 transfectants with empty vector were used as a negative control (Mock). (B) S100A14 was secreted extracellularly from KYSE180 cells whereas no S100A14 secretion could be detected in EC9706 cells. (TIF) [file pone.0147881.s001.tif]
